# Quantifying the contributions of climate change and adaptation to mortality from unprecedented extreme heat events

**DOI:** 10.1073/pnas.2503577122

**Published:** 2025-12-16

**Authors:** Christopher W. Callahan, Jared T. Trok, Andrew J. Wilson, Carlos F. Gould, Sam Heft-Neal, Marshall Burke, Noah S. Diffenbaugh

**Affiliations:** ^a^Department of Earth System Science, Stanford University, Stanford, CA 94305; ^b^Center on Food Security and the Environment, Stanford University, Stanford, CA 94305; ^c^School of Public Health, University of California San Diego, La Jolla, CA 92093; ^d^Department of Environmental Social Sciences, Stanford University, Stanford, CA 94305; ^e^National Bureau of Economic Research, Cambridge, MA 02138; ^f^Woods Institute for the Environment, Stanford University, Stanford, CA 94305

**Keywords:** climate attribution, extreme heat, health impacts

## Abstract

Understanding the mortality effects of the most extreme heat events is central to climate change risk analysis and adaptation decision-making. Accurate representation of these impacts requires accounting for the effects of prolonged sequences of hot days on mortality, the change in that mortality due to anthropogenic forcing, and the potential compensating effects of adaptation to heat. Here, we revisit the August 2003 heat wave in France, a canonical event in a region with rich climate and mortality data, to understand these influences. We find that standard heat–mortality exposure–response functions underpredict excess deaths in August 2003 by 55% but that accounting for the temporally compounding effects of hot days better matches observed mortality. After accounting for compounding effects and applying a machine learning approach to single-event climate attribution, we attribute 6,079 deaths in August 2003 to climate change. Finally, we show that recent adaptation to heat in France has reduced the projected death tolls of future 2003-like events by more than 75%.

## Significance

We revisit the August 2003 heat wave in France to better understand the influences of climate change and human adaptation on mortality from unprecedented extreme heat events. We reconfirm previous findings of approximately 16,000 excess deaths during the event, but show that standard heat–mortality exposure–response functions underestimate this death toll by 55% in an out-of-sample test. Using an exposure–response function accounting for consecutive hot days that more effectively predicts mortality in 2003, we attribute more than 6,000 deaths in August 2003 to climate change. Additionally, we show that the heat–mortality relationship has moderated since 2003. As a result, we project future 2003-like events to cause 77% fewer deaths in France compared to a world without that moderation.

Understanding the contribution of climate change to human mortality from unprecedented extreme heat events is a critical priority (1). Unprecedented extreme climate events can stress infrastructure or adaptation measures that have been benchmarked to recent experience, posing challenges for societal resilience (2). These record-breaking events are increasing due to anthropogenic forcing (3–5), with future climate change likely to generate even more extreme events than those that have been recently witnessed (6). With respect to historical events, attributing the observed health impacts of extreme heat to climate change (7–10) has the potential to inform ongoing climate litigation (11, 12) and loss and damage compensation (13). With respect to the future, evaluating the impacts of previously unseen events made newly possible by global warming is essential to understanding the health risks of future global temperature levels (14, 15) and informing cost–benefit analysis tools such as the social cost of greenhouse gases (16, 17).

At the same time, people have a well-documented ability to adapt to extreme weather, often leading to reductions over time in the effect of heat exposure on mortality (18–23). If the conditions that generated historical extreme heat events recur at present or future levels of warming (15), they may occur not only in a different climate context but also against the backdrop of an evolving temperature–mortality relationship. As a result, accurately quantifying the past and future health risks of extreme heat requires evaluating the competing influences of climate warming and adaptation on mortality.

However, characterizing the impacts of unprecedented extreme events poses specific analytical challenges. Empirically derived exposure–response functions are a standard tool to quantify the health impacts of climate change but are estimated using data that by definition do not include unprecedented future events. Further, these models tend to treat hot days as additively separable predictors of mortality, neglecting potential compounding effects of multiple days in sequence. Multiple hot days may result in heat accumulation in both the built environment and human bodies (24), and mortality during previous unprecedented events may have been driven by sequences of warm nights that prevented people from cooling themselves after hot days (25). On the other hand, many statistical studies find little additional effect of the sequencing of hot days above their independent effects (26–28). As a result, it remains unclear whether standard statistical models estimated on the full historical distribution of temperatures are suitable for quantifying the effects of unprecedented sequences of very hot temperatures.

Here, we revisit the August 2003 heat wave in France, an event which offers insight into the contributions of climate change and adaptation to extreme heat mortality for multiple reasons. First, when it occurred, 2003 was the hottest summer in Europe in at least the previous 500 y (29, 30) and the level of heat that was reached was partly due to climate change (31), making 2003 a useful test case for events that are out-of-sample relative to recent experience. Second, mortality in France appeared to be uniquely sensitive to heat prior to 2003 (19), yielding severe mortality during this event (32–34). For example, many of the victims lived in small, poorly ventilated apartments directly under zinc roofs that were extremely effective at trapping heat, potentially exposing residents to the compounding effects of multiple days of heat accumulation (35, 36). Third, France collects detailed daily mortality data, enabling robust statistical analysis. Finally, France adopted a series of adaptation measures immediately following the 2003 event, including the expansion of air conditioning in vulnerable locations such as nursing homes (18) and heat action plans that include educational messaging and proactive visits to isolated people during hot periods (37). Comparing the temperature–mortality relationship before and after 2003 thus offers a simple way to assess the effectiveness of these adaptation measures and other societal changes (18–22).

We take five key steps in our analysis (*SI Appendix*, Fig. S1). First, we evaluate the skill of standard exposure–response functions when applied out-of-sample to the 2003 event, to determine whether empirically derived functions can skillfully represent the impacts of unseen events. Second, we develop response functions that explicitly incorporate temporally compounding heat. Third, we combine these exposure–response functions with a machine-learning-based approach to extreme climate event attribution (38) to quantify the contribution of climate change to mortality in August 2003. Fourth, we evaluate the change in exposure–response functions before and after 2003 and quantify the effect of adaptation on heat-related mortality in the recent period. Finally, using the same machine-learning-based approach to project the intensity of 2003-like events if such events were to recur in a warmer climate, we quantify the extent to which recent adaptation can offset the mortality impacts of increasingly intense future heat events.

## Mortality During August 2003

**Fig. 1.**
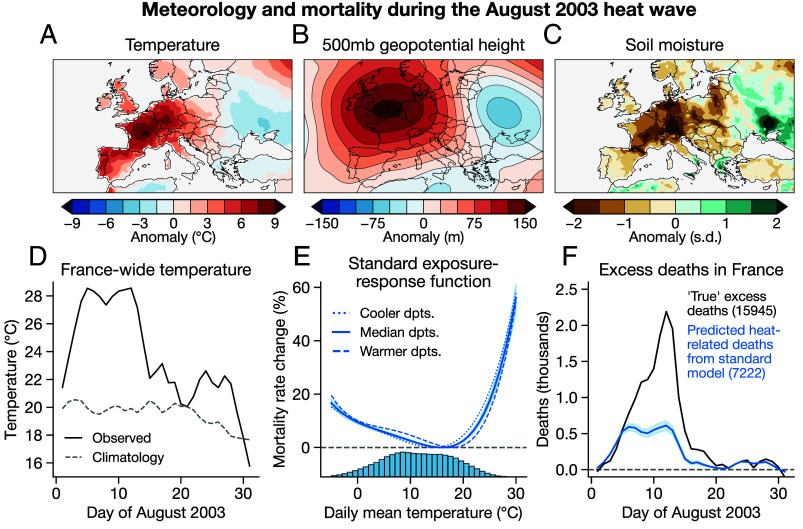
Physical and epidemiological characteristics of the August 2003 heat wave in Europe. (*A*–*C*) Anomalies of temperature (*A*), 500-mb geopotential height (*B*), and soil moisture (*C*) averaged over 1–14 August 2003. (*D*) Population-weighted average temperature across French departments in August 2003 (black), along with the 1980–2002 mean (gray, dashed). (*E*) Exposure–response function relating daily mean temperature to mortality rates over 1980–2002, using a fourth-order polynomial, five lags of temperature, and a continuous interaction with department mean temperature (*Materials and Methods*). Different lines show the response functions at the three terciles of department mean climate. The lower histogram shows distribution of daily temperatures in the sample. (*F*) Excess deaths (black) and heat-related deaths (blue) across France. Excess deaths are based on deviations relative to averages and heat-related deaths are based on the exposure–response function in panel (*E*). In (*E* and *F*), shading shows 95% CIs.

The first two weeks of August 2003 were characterized by extreme temperatures centered on France, Germany, and Spain (Fig. 1*A*), a high-pressure system centered north of France (Fig. 1*B*), and dry soils across much of the continent (Fig. 1*C*). Temperatures across France peaked at the end of the first week of August and through the second week, with a peak of 28.6 °C (daily mean) on 12 August (Fig. 1*D*).

To understand the death toll of this event, we first derive standard exposure–response functions that relate daily temperatures in French administrative regions (*“départements”* or “departments”) to mortality in those regions, accounting for spatial heterogeneity as a function of baseline climate (*Materials and Methods*). We use a fourth-order polynomial in daily mean temperature across each day and the 5 d following it to account for lagged responses to temperature (*Materials and Methods*). We find a strong nonlinear relationship between temperature and mortality, where mortality rates increase at both cold and hot temperatures (Fig. 1*E*). We also observe spatial heterogeneity in this relationship that may suggest some baseline adaptation to temperature, with warmer departments exhibiting a slightly weaker response to high temperatures (Fig. 1*E*). Our findings are similar to the responses found in many previous studies, including those using two-stage pooled time series models (39). They are also similar when we use different numbers of lags, polynomial orders, fixed effects, or temperature variables (*SI Appendix*, Fig. S2).

To assess the “true” death toll in August 2003, we also calculate excess deaths relative to region- and time-specific baselines (*Materials and Methods*). Excess deaths are a standard epidemiological approach to quantify elevated mortality without specifying a cause of death or parametric exposure–response function. We estimate ∼15,900 excess deaths in France across all of August (Fig. 1*F*), which aligns well with other estimates (32). Note that hereafter we use “excess deaths” to refer to estimates of total elevated mortality relative to averages and “heat-related deaths” to refer to mortality predicted by a temperature exposure–response function.

The standard exposure–response model shown in Fig. 1*E* is estimated using data from 1980 through 2002, but not including 2003, so we can perform an out-of-sample prediction of heat-related mortality during August 2003 and compare it to our estimate of excess deaths. Using this standard temperature–mortality association (Fig. 1*E*) to predict the August 2003 death toll underestimates total mortality by 55%: 7,222 heat-related deaths (95% CI: 6,494–7,917) compared to 15,945 excess deaths (Fig. 1*F*). This underestimate is not unique to our specification; alternative polynomials, fixed effects, lag lengths, and temperature exposures yield similar results (*SI Appendix*, Fig. S3).

Because excess deaths do not specify a cause or exposure–response function, an alternative interpretation of this result is that there were only ∼7,200 heat-related deaths and some other cause explains the remaining ∼8,700. However, there is no other known cause concurrent with the heat wave that would explain such a large number of excess deaths (35), and the magnitude of the mortality increase far exceeded typical variation from other causes (32). We thus interpret the gap between excess deaths and heat-related deaths as an underestimate from the exposure–response function rather than an overestimate from the excess deaths calculation.

## Temporally Compounding Heat-Related Mortality

We hypothesize that neglecting the unique effects of multiple hot days in sequence may contribute to the underestimate from the standard model, which does not consider the order of hot days within our 5 d of lags. To incorporate temporal compounding, we modify our regression to distinguish between hot days that occur in sequence and hot days that occur after typical or cool days. Specifically, again using the pre-2003 data, we estimate response functions where the current day’s temperature is interacted with the previous day’s temperature. This specification allows the effect of current-day temperatures on mortality to vary according to the previous day (*Materials and Methods*). We include a separate interaction with department mean climate to allow spatial heterogeneity, as in the standard model (Fig. 1*E*); this interaction is included in all calculations, but we present results evaluated at the population-weighted average department temperature in the text and figures for clarity.

**Fig. 2.**
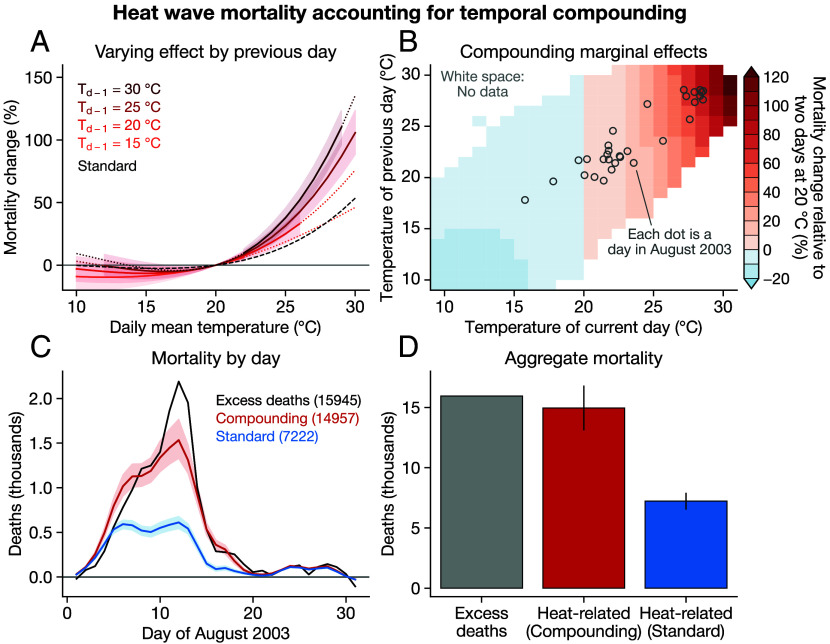
Temporal compounding of heat mortality. (*A*) Mortality exposure–response functions varying by the temperature of the previous day. CIs are only shaded in the range of current-day temperatures that occur given the previous day’s temperature, and lines are dotted when extrapolating outside of that range. The black dashed curve indicates the “standard” exposure–response function from Fig. 1*E*. Each model also includes a continuous interaction with department mean temperature and is shown at the population-weighted average department temperature for simplicity. (*B*) Two-dimensional surface of marginal effects, illustrating the effect of combinations of current days (x-axis) and previous days (y-axis), compared to two consecutive days at 20 °C. Cells are not colored if that combination of current and previous days does not occur in the pre-2003 data. Gray dots show population-weighted France-wide temperatures on each day of August 2003. (*C*) Predicted mortality from the heat wave by day from both the compounding and standard models. The solid line shows mean and shading shows 95% range. (*D*) Total heat-related and excess deaths in August 2003. The bar height shows average prediction and the black line shows 95% range.

We find that, before 2003, sequencing a hot day after another hot day can nearly double the mortality effect of that hot day relative to if it occurred in isolation (Fig. 2 *A* and *B*). For the average department, a 30 °C day increases mortality by 76% if it follows a 20 °C day, relative to two days at 20 °C (Fig. 2*B*). However, when a 30 °C day follows another 30 °C day, the mortality increase rises to 136% (Fig. 2*B*).

The strong differentiation between the responses conditional on previous days suggests an important role for temporal compounding in shaping heat wave mortality. Indeed, predicting August 2003 mortality using the compounding model yields a death toll that is closer to the total excess deaths than the standard model (Fig. 2*C*). The mean prediction from the compounding model is 14,957 heat-related deaths (CI: 13,350–16,810) compared to the mean of 7,222 from the standard model, and the CIs from the compounding model include the total excess deaths value of 15,945 (Fig. 2*D*).

While the skill of our predictions is increased relative to the standard model, our predictions still underestimate peak daily mortality, especially on 12 and 13 August (Fig. 2*C*), yielding an underestimate of total mortality throughout the month (Fig. 2*D*).

To evaluate the robustness of the effect of temporal compounding, we test two alternative approaches for the interaction between current and previous days, and find that both yield very similar results (*Materials and Methods*). One factor that slightly affects the results is the number of lags in the regression. We use 5 lags because the effects of heat appear to accrue only in the first several days after exposure (*SI Appendix*, Figs. S4 and S5), though the effects of cold days take longer (*SI Appendix*, Fig. S4) (40). Using 10 lags in the compounding model yields generally similar results, though with a notable overestimate of mortality in the third week of August after the peak of the heat (*SI Appendix*, Fig. S6), yielding an overestimate of aggregate event mortality (17,998 heat-related deaths vs. 15,945 excess deaths).

## Global Warming Contributions to Heat-Related Mortality

**Fig. 3.**
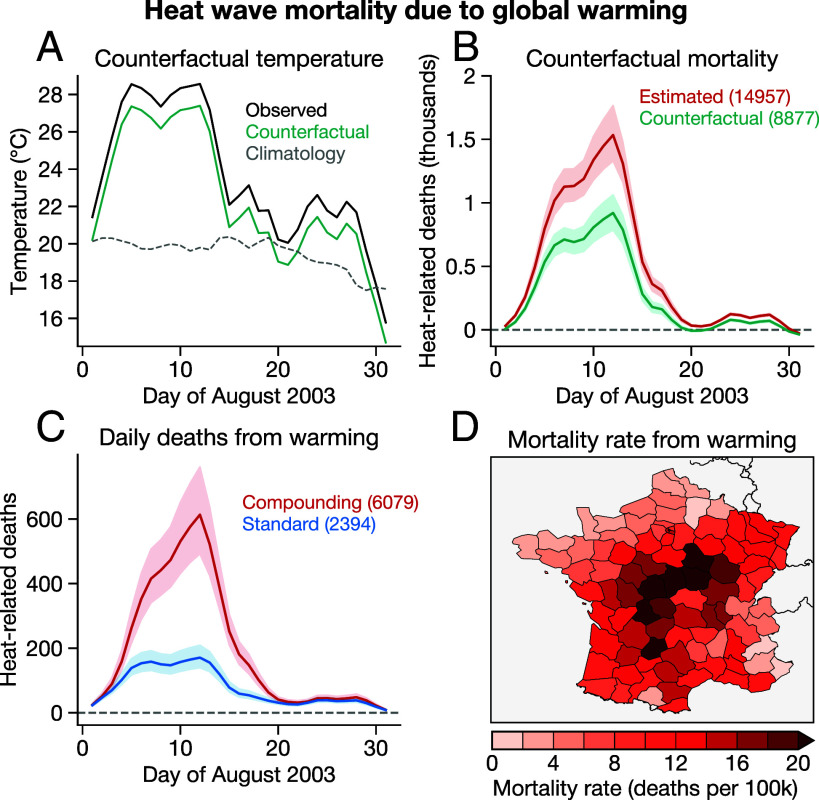
Mortality attributable to global warming in August 2003. (*A*) Observed (black), counterfactual (green), and climatological (gray) temperatures across France in August 2003. (*B*) Mortality in August 2003 under observed conditions (red, as in Fig. 2*D*) and under counterfactual conditions (green). (*C*) Mortality attributable to climate change, calculated as the predictions from observed conditions minus the predictions from counterfactual conditions. The blue curve shows the analogous calculation using the standard model. In (*B* and *C*), the solid line shows the mean prediction and shading shows 95% range. (*D*) Mortality rate due to climate change in each French department, defined as deaths per 100,000 population.

An improved representation of the underlying heat–mortality response allows us to return to the question of how many heat-related deaths in August 2003 were due to increasing global average temperature. We use convolutional neural networks trained on global climate models to simulate counterfactual August 2003 temperatures at 0 °C of global mean temperature change, rather than the ∼0.8 °C observed at the time of the event (*Materials and Methods*), as the historical increase in GMT has been driven by human emissions of greenhouse gases (41). We find that climate change increased temperatures across France by an average of 1.2 °C in the first two weeks of August 2003 (Fig. 3*A*).

Applying our exposure–response functions to these observed and counterfactual temperatures, we find that the counterfactual event in the absence of global warming would have caused 8,877 excess deaths, rather than the 14,957 we estimate occurred with observed temperatures (Fig. 3*B*). As a result, we attribute 6,079 heat-related deaths to climate change (CI: 5,043–7,373), 41% of the mortality from the event and more than double the analogous result from the standard model that does not account for temporal compounding (Fig. 3*C*). Climate change-driven mortality was substantial across all of France but concentrated in the center of the country (Fig. 3*D*), with >20 deaths per 100,000 people contributed by global warming in some departments.

The contribution of climate change to mortality witnessed in 2003 raises the question of the mortality from a similar event if it occurred in the near future. To answer this question, it is important to consider that France may have adapted to heat extremes following 2003 by adopting measures such as heat action plans (37), potentially altering the future death toll of a physically similar event (18). To test this question, we re-estimate the exposure–response function using data from 2004 to 2019, under the assumption that shifts in the response over time indicate adaptation.

**Fig. 4.**
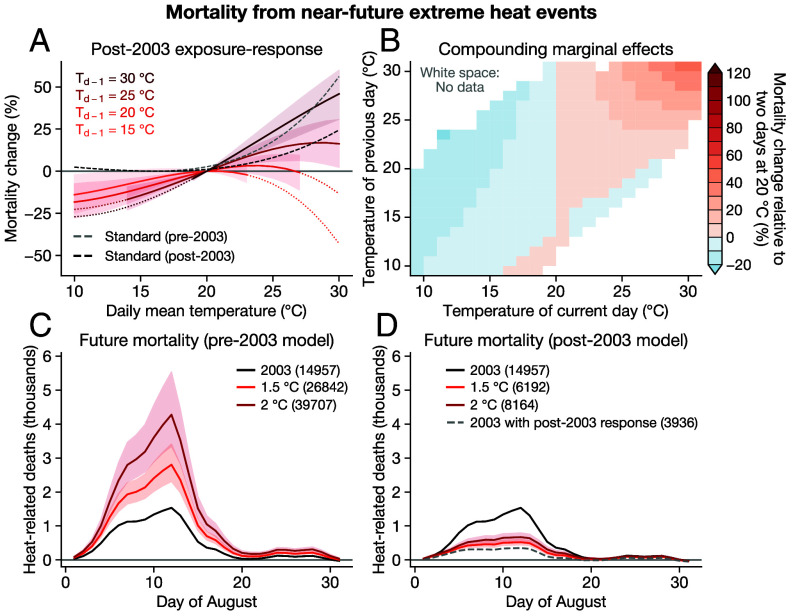
Projected mortality from 2003-like events in the near future. (*A*) Exposure–response functions as in Fig. 2 using the standard approach (black dashed line) and accounting for temporal compounding (red lines), fitted to data from 2004 to 2019 instead of 1980 to 2002. The gray dashed line shows the standard model from the 1980–2002 sample (as in Fig. 2*A*). CIs are only shaded in the range of current-day temperatures that occur given the previous day’s temperature, and lines are dotted when extrapolating outside of that range. (*B*) Two-dimensional marginal effects as in Fig. 2*B*, but for the 2004–2019 sample. Colorbar is the same as Fig. 2*B*. (*C*) Predicted heat-related deaths for a 2003-like event at annual global mean temperature anomalies of 1.5 °C and 2 °C using the 1980–2002 (i.e., pre-2003-event) exposure–response functions. The black line shows the prediction for 2003 at its original temperature. All predictions use the model incorporating temporal compounding. (*D*) As in (*C*), but using the 2004–2019 exposure–response functions. The gray dashed line shows predictions using 2003 temperatures but with the post-2003 exposure–response functions. In (*C* and *D*), the solid line indicates mean projection and shading shows 95% range.

The response of mortality to temperature in 2004–2019 is milder than in 1980–2002 (Fig. 4*A*); in the later period using the standard model, a 30 °C day vs. a 20 °C day increases mortality by 23% for the average department, compared to 54% in the earlier period. And while there remains differentiation between the effects of hot days following previous hot or mild days, the compounding model yields results that are similarly muted compared to before 2003. Consecutive 30 °C days increase mortality by 46% compared to consecutive 20 °C days, a 66% reduction compared to the analogous figure of 136% before 2003 (Fig. 4*B*).

We observe small apparent protective effects of high temperatures for some combinations of current and previous days (Fig. 4 *A* and *B*), likely a result of extrapolating a polynomial to regions where there are few or no data. The CIs on these responses contain zero, and the standard model still shows mortality increases at high temperatures (Fig. 4*A*), so in the aggregate high temperatures remain dangerous even after 2003.

Our counterfactual simulations are based on projecting the same meteorological conditions (Fig. 1*A*–*C*), but at higher levels of annual global mean temperature (GMT; *Materials and Methods*). We use annual GMT anomaly values of 1.5 °C and 2 °C for present and near-future conditions, respectively, emphasizing that these values refer to annual GMT rather than long-term global warming levels (42, 43). (For reference, the annual GMT anomaly was ∼0.8 °C in 2003 and ∼1.5 °C in 2024).

If the 2003 meteorological conditions were to occur at global temperature levels of 1.5 °C or 2 °C but with the pre-2003 exposure–response function, we estimate heat-related mortality of 26,842 and 39,707 deaths, respectively (Fig. 4*C*). That is, if the temperature–mortality relationship had not changed following 2003, we estimate that near-term warming could approximately double the death toll witnessed in 2003. On the other hand, incorporating post-2003 changes in the response function reduces the projected death toll by nearly an order of magnitude (Fig. 4*D*), with excess deaths of 6,192 and 8,164 at 1.5 °C and 2 °C. In other words, France’s lower post-2003 sensitivity to extreme heat has reduced the death toll of a future 2003-like event by 77%. Similarly, if these adaptations had been undertaken prior to 2003, we calculate that the August 2003 death toll would have been 3,936, a ∼74% reduction (Fig. 4*D*).

However, we emphasize that even with this adaptation, a 2003-like event at 1.5 °C annual GMT would, at its peak, increase daily mortality by 38% above its average daily rate. This is substantially lower than the 111% increase estimated during the peak of the 2003 event, but nevertheless highlights the magnitude of the health impacts of extreme heat, even after substantial adaptation.

## Discussion

Our results demonstrate that during at least one widely studied extreme event, the sequencing of multiple extreme hot days played a key role in driving heat-related mortality. This finding differs from other work arguing that hot days have an additively separable effect on health (26–28). However, given that temporal compounding appears to play a smaller role following 2003, our results may not generalize to all events, especially since pre-2003 France appeared to be an outlier in its heat sensitivity even relative to the rest of Europe (19). Still, given projected future increases in record-shattering heat events (6), understanding the impacts of sequences of hot days in other regions remains a research priority.

Our estimate of >6,000 deaths attributable to climate change in August 2003 in France is larger than the previous estimate by Mitchell et al. (7) of 506. There are several differences between our studies worth exploring. First, we use an exposure–response function that predicts greater heat-related mortality during the event. Second, we calculate mortality across all of France rather than just in Paris. Third, they attribute 70% of the event mortality to climate change based on the Fraction of Attributable Risk (FAR) for the event, rather than the 41% we find based on the contribution of climate change to the event magnitude. More recent research has shown that because the FAR measures the change in the event’s probability, rather than its magnitude, it is often not appropriate for impact attribution (10); however, attribution of 2003 mortality has yet not been revisited since this recognition. Finally, we focus just on August 2003 rather than June, July, and August, although this likely has a small effect since the vast majority of heat-related mortality occurred in August (7).

One way to provide a more direct comparison between our results and Mitchell et al. (7) is to limit our analysis to Paris specifically. In Paris, we attribute 397 deaths to climate change over August 2003, smaller than Mitchell et al.’s value of 506. However, if we rescale our estimate to attribute 70% of the event mortality to climate change instead of 41%, we would attribute 687 deaths in Paris to warming, which is 36% greater than the estimate of Mitchell et al. This calculation illustrates how attribution of human impacts to climate change is a function of both the calculation of the climate change contribution to a physical hazard and the model used to connect that hazard to human outcomes.

One inherent tension in our attribution analysis is that it requires imagining a heat wave at preindustrial global temperatures but 2003 levels of economic development and societal sensitivity to heat. In other words, we separate the climate consequences of fossil-fueled energy use from the economic and social consequences (the latter of which include a number of benefits). While we have well-established physical understanding for how an observed heat wave would have manifested without global warming, understanding of how adaptation to heat has changed over the past several hundred years is more limited. For example, it is unclear whether currently observed sensitivity to heat is driven by long-term exposure to warm baseline climates (44), more recent innovations in air conditioning enabled by economic growth (21), or some combination of the two (17). Additionally, there is mixed evidence regarding whether the sensitivity of mortality to heat has declined over the last several decades (19). As a result, it is unclear whether a counterfactual heat wave in a cooler climate would also have been accompanied by a different mortality response to that heat.

While this tension is present in most similar attribution studies, there are opportunities for progress. For example, it is possible to model counterfactual future heat waves alongside evolving societal sensitivity to heat by treating present-day locations with a given climate and income as surrogates for different places with the same characteristics in unobserved periods (15, 17). Additionally, it is possible to quantify adaptation by studying the effectiveness of particular interventions at modifying exposure–response relationships (44), rather than simply describing aggregate shifts in those relationships. Both of these approaches, and others, provide opportunities to more comprehensively understand the coevolution of climate and society as global temperatures rise.

Despite these challenges, in our setting, we do find strong evidence that the post-2003 response function is very different from the pre-2003 response (Fig. 4*A*). This may reflect adaptations undertaken in response to the death toll in 2003, as has been suggested by other analyses (18, 22, 37). Indeed, when we simulate the same heat event with two different response functions (Fig. 4 *B* and *C*), we find that the milder response function generates a ten-fold reduction in mortality, suggesting large health benefits from these adaptations. However, many other countries have not adopted the same measures as France (45), and adaptation to extreme heat appears limited on a global scale (19). So, progress in France may not indicate widespread adaptation elsewhere, though it does help to quantify the potential benefits of adaptation in the context of intensifying heat extremes.

It is notable that our model of temporal compounding underestimates daily deaths at the peak of the event (Fig. 2*C*). There are a number of social factors that may have contributed to the extreme mortality peak, such as overstretched health infrastructure resulting both from the August vacation in France and from enormous demand for health care during the event (35, 46). Given that our models are estimated using data only prior to 2003, which may not include similar pressures on the health system, they may not be entirely effective at capturing the precise temporal dynamics of the most extreme heat events.

An additional factor that may have contributed to the elevated peak of mortality is air pollution, which we do not consider explicitly. Ozone concentrations were high during the 2003 heat wave, potentially contributing to mortality (47, 48). However, spatially explicit daily ozone data are limited over our time period of interest, making it difficult to directly model the effect of pollution on mortality. Further, high temperatures can contribute to increases in air pollution (49), so controlling for pollution concentrations in our statistical models may inappropriately exclude an important pathway by which temperature affects mortality. Understanding the dynamic interactions between temperature, air pollution, and mortality is an important area of active research.

Ours is an analysis of opportunity in some respects. France makes local daily mortality publicly available, but many other governments either do not collect or do not share comparable data, posing a challenge for researchers. Further, our machine-learning-based attribution approach has been shown to skillfully simulate temperature during the August 2003 event, but other heat waves such as the 2021 Pacific Northwest event have proven difficult to simulate both by our method (38) and others (50, 51). Alongside more sophisticated exposure–response functions, additional advances in physical event attribution may be necessary to understand the climate change contribution to mortality during more recent unprecedented events.

## Conclusion

Our analysis uses econometric and machine learning tools to reveal several important insights about the influences of climate change and adaptation on mortality during the canonical 2003 heat wave in France. After accounting for the effects of multiple hot days in sequence, climate change can be linked to around 40% of the mortality of this event, even though the GMT anomaly was only approximately half of its current value (0.8 °C in 2003 vs. 1.5 °C in 2024). Even if global temperatures are stabilized near their current levels, this additional warmth may contribute more than half of mortality during future similar extreme heat events (15). However, we also reveal significant potential to reduce these harms if strong adaptation actions are taken. Widespread adoption of policies similar to those undertaken in France following 2003 may be necessary to avert mass mortality from future extreme heat.

## Materials and Methods

### Data.

Our primary climate data is from the E-OBS station-based data product (52), which we use in the regression models and mortality prediction. E-OBS data are aggregated to the level of French departments, weighting by the population of each grid cell within the department. To plot the maps in Fig. 1, we use ERA5 reanalysis data (53) averaged over 1–14 August, with anomalies defined relative to grid cell and day of year.

Daily mortality data spanning 1980–2019 on the universe of deaths in France are made available by INSEE, the French statistical agency (https://www.insee.fr/fr/information/4769950). These data are provided at the commune level, a relatively fine geographic resolution, but we aggregate them to departments for comparison with climate data and to merge them with population data. We drop overseas territories from this analysis and focus only on the 94 departments in continental France.

### Excess Mortality.

Excess mortality is a standard calculation to assess deviations in mortality from expected conditions. The procedure is twofold: 1) model mortality as a function of spatial and temporal baseline factors; and 2) subtract these baseline values from observed mortality during some time period of interest.

We model all-age, all-cause mortality over 1980–2019 as a function of department-specific day-of-year averages and department-specific annual averages, allowing each department to have its own seasonal cycle and long-term trend. Specifically, we estimate an Ordinary Least Squares model for the log of the mortality rate (M) in department i as a function of day-of-year d and year y:

[1] $$\begin{array}{c} \log\left(M_{\mathrm{idy}}\right)=\mu_{\mathrm{iy}}+\delta_{\mathrm{id}}+\epsilon_{\mathrm{idy}} \end{array}$$

For each department-day in August 2003, we then subtract the predicted values using this equation from the observed mortality rate to calculate excess mortality.

### Standard Temperature–Mortality Exposure–Response Function.

The goal of an exposure–response function is to describe a relationship between an exposure (e.g., temperature) and an outcome of interest (e.g., mortality rates). A standard approach is to regress mortality rates on a function of temperature, usually nonlinear, as well as nonparametric controls for all region- and time-specific average factors that could confound this relationship (“fixed effects”). Our approach models log mortality rates as a function of local daily temperature, department-by-day-of-year fixed effects, and department-by-year fixed effects:

[2] $$\begin{array}{cc} \log(M_{\mathrm{idy}})= & \sum_{j=0}^{L}\left[f(T_{i(d-j)y}+f\left(T_{i(d-j)y}\right)\times {\bar{T}}_{i}\right]\\ & +\sum_{j=0}^{L}\left[{\vec{\lambda }}_{j}{\vec{X}}_{i(d-j)y}\right]+\mu_{\mathrm{iy}}+\delta_{\mathrm{id}}+\epsilon_{\mathrm{idy}} \end{array}$$

We note several features of this equation. First, the fixed effects ($\mu_{\mathrm{iy}}+\delta_{\mathrm{id}}$) are the same as those used in the excess mortality estimation (Eq. **1**). In effect, then, this approach seeks to isolate the temperature-driven component of excess mortality.

Second, we include L lags of daily temperature to account for the delayed effects of heat and cold. In our main analysis, we use 5 lags, since the effect of heat appears to occur primarily in the first several days and to decay well before the fifth day later (*SI Appendix*, Fig. S4). (We test the sensitivity of this choice by extending the lags to 10 and 30 or reducing them to 3; *SI Appendix*, Figs. S2 and S3.) We estimate independent coefficients for each daily lag of temperature, rather than specifying a particular parametric fit across the lags. This simple distributed lag approach is similar to that used in other papers on temperature and mortality (40) but does differ from other work that applies a smooth function or spline across the lag dimension (39). Because the effect of extreme heat on mortality only occurs within the first 3 to 4 d after exposure (*SI Appendix*, Fig. S4), a more sophisticated specification across the lag dimension appears unnecessary in our setting.

Third, we interact daily temperatures with each department’s long-term mean temperature (${\bar{T}}_{i}$) to allow the response function to vary over space. Different locations might make different investments in adaptation resources based on their climate; for example, Mediterranean regions of France may be more likely to invest in air conditioning since they are warmer on average (22). Interacting a nonlinear function of temperature with long-term climate is a standard approach to account for spatial heterogeneity (17, 54, 55).

Finally, $\vec{X}$ refers to a vector of controls that we include alongside temperature, specifically daily mean relative humidity and daily accumulated precipitation.

In our main analysis, we use a fourth-order polynomial for f(·) and daily mean temperature for the exposure variable, both following other work (17, 19). Daily mean temperature, relative to daily maximum or minimum, has the advantage of balancing the effects of both hot days and warm nights, which aligns with our interest in the effects of heat accumulation. In *SI Appendix*, Fig. S2, we show results using a cubic model or daily maximum or minimum temperature, which are qualitatively similar to our preferred specification.

### Incorporating Temporal Compounding.

As with most other work in this domain, the exposure–response function in Eq. **2** treats temperatures on different days as linearly additive. Two hot days have the same effect on mortality if they occur on d−5 and d or if they occur on d−1 and d. Our approach to temporal compounding is to relax this assumption by interacting the temperature on each day with the temperature of the previous day. This approach asks the question: Does a hot day have the same effect when it occurs after another hot day as when it occurs after an average or cold day?

We estimate several variations of the following model:

[3] $$\begin{array}{cc} \log(M_{\mathrm{idy}})= & \sum_{j=0}^{L}\left[f(T_{i(d-j)y}+f\left(T_{i(d-j)y}\right)\times g\left(T_{i(d-j-1)y}\right)\right]\\ & +\sum_{j=0}^{L}\left[f\left(T_{i(d-j)y}\right)\times {\bar{T}}_{i}+{\vec{\lambda }}_{j}{\vec{X}}_{i(d-j)y}\right]+\mu_{\mathrm{iy}}+\delta_{\mathrm{id}}+\epsilon_{\mathrm{idy}} \end{array}$$

This model adds the interaction with temperatures on day d with temperature on day d−1. For lagged days d−j, we interact that day’s temperature with the temperature on day d−j−1. For example, temperature on day d−2 is interacted with temperature on day d−3.

The other components of this regression equation are the same as the standard model, including the fixed effects, the interaction with mean temperature, and the controls for humidity and rainfall.

The temperatures of a day and the day preceding it are not independent, statistically or physically. *SI Appendix*, Fig. S7 shows a strong correlation between the temperature on day d−1 and the temperature on day d. While this correlation does not prevent us from identifying the effect of temporal compounding, it does mean there are many combinations of days that do not appear in the data (e.g., a 10 °C day followed by a 30 °C day does not occur in the data; see white space in Fig. 2*B*). An alternative approach is to interact temperature on day d with temperature *anomalies* on day d−1, which are less strongly correlated and may therefore provide increased statistical power (*SI Appendix*, Fig. S7). Reproducing our predictions for August 2003 using this approach yields very similar results, with aggregate mortality of ∼14,000 deaths (*SI Appendix*, Fig. S6).

An additional concern is that our main model specifies a linear interaction between days, when the true relationship may be nonlinear. To test this assumption, we re-estimate the compounding model using natural cubic spline in the previous day’s temperature, with a knot at the sample-wide median daily temperature of ∼11.7 °C. This approach allows separate nonlinear interactions for cold previous days and hot previous days and again yields extremely similar results to our main analysis (*SI Appendix*, Fig. S6).

In all regressions, we sample uncertainty in exposure–responses by generating a multivariate normal distribution of each vector of coefficients (n=500) based on the variance–covariance matrix of each model (17). We cluster SEs by department to account for autocorrelation in mortality. In the figures and text, we present the mean and 95% CI (2.5th to 97.5th percentiles) of these 500 samples.

### Machine Learning Predictions.

We use the machine-learning-based extreme event attribution method developed by Trok et al. (38). This approach trains convolutional neural networks (CNNs) on an ensemble of climate model simulations to predict regional daily temperature from the daily meteorological conditions (including geopotential height, surface pressure, and soil moisture), the calendar day, and the annual global mean temperature (GMT) anomaly in the year leading up to the event. The trained networks are then applied to ERA5 reanalysis in an out-of-sample prediction to predict regional temperatures as a function of observed meteorological conditions. Trok et al. (38) showed that these predictions closely reproduce observed daily temperatures during the 2003 event.

Because this approach takes meteorology, day of year, and GMT as separate predictors, we can use the trained models to create new predictions which maintain the same meteorology and day of year, but prescribe an alternative GMT. We prescribe annual GMT anomalies of 0 °C for the counterfactual predictions without human influence (Fig. 3), given the strong evidence that the historical increase in GMT has been driven by human influence on the climate system (41). For the near-future events (Fig. 4), we prescribe annual GMT anomalies of 1.5 °C and 2 °C.

The results of this prediction procedure are “counterfactual” events, quantifying the regional temperature that would have resulted from the same meteorological conditions if they had occurred during a preindustrial or future climate instead of the actual climate of 2003. While our experimental setup uses machine learning to create predictions of historical events under different global temperature conditions, our approach is a version of previous “storyline” event attribution studies that distinguish between the meteorological drivers of extreme events and the human contribution to increasing the intensity of those events (56, 57).

The target for the predictions is the average temperature over a region in southern and central Europe that encompasses France, exactly as in ref. 38. We train the CNNs on three realizations each of five climate models from the sixth phase of the Coupled Model Intercomparison Project (CanESM5, UKESM1-0-LL, HadGEM3-GC31-LL, MIROC6, and MPI-ESM1-2-LR). Because this machine learning methodology requires relatively extensive data—daily data from three-dimensional meteorological variables for multiple realizations from a given climate model—these five models are the only ones currently available for training. The CNN is trained across the pooled sample of climate models, but includes an indicator variable for the model, allowing us to make separate predictions for each model. In training, we also vary the random seed 10 times to account for randomness in the training process. One final change we make to the method developed by Trok et al. is to use daily mean temperature as the predictand instead of daily maximum temperature.

We use a simple delta-method bias correction before applying these predictions to our observational data. First, for each day in August 2003, we take the difference between the predictions at the counterfactual global temperatures (0, 1.5, and 2 °C) and the predictions at the observed global temperature (0.8 °C in 2003). We then apply the region-wide “delta” for each day uniformly to each department’s temperature for that day to create counterfactual temperature time series for each department.

### Calculating Heat-Related Mortality.

We calculate heat-related mortality by applying exposure–response functions to observed or counterfactual time series and climatological baselines. Because the dependent variable in the regressions is log mortality, comparing the function of two different temperature values yields percent changes in mortality. We then multiply these percent changes in mortality by baseline numbers of deaths to calculate additional deaths due to heat.

When calculating mortality from the 2003 event, we compare observed temperatures in 2003 to the 1980–2002 average for each corresponding calendar day, and multiply the resulting percent differences by the 1980–2002 average number of deaths for each calendar day.

For calculations of counterfactual heat-related mortality, we combine the 500 samples of the regression coefficients and the 50 different machine learning predictions (the CNN makes separate predictions for each of five climate models across each of ten random seeds), yielding 25,000 total estimates. In our main analysis, we simply pool all CNNs trained on the different climate models. The different climate models yield slightly different estimates, but all are qualitatively similar (*SI Appendix*, Fig. S8).

## Supplementary Material

Appendix 01 (PDF)

## Data Availability

Data and code required to reproduce the findings of this study are available at: https://zenodo.org/records/16953793 (58).
