# Supplementary material for: Quantifying the contributions of climate change and adaptation to mortality from unprecedented extreme heat events
Source: Proc Natl Acad Sci U S A. 2025 Dec 16;122(51):e2503577122. doi: 10.1073/pnas.2503577122 (PMC12745773; doi:10.1073/pnas.2503577122)
Supplement: Supplementary file 1 — Appendix 01 (PDF) [file pnas.2503577122.sapp.pdf]

Supporting Information for

Quantifying the contributions of climate change and adaptation to mortality from unprecedented extreme heat events

Christopher W. Callahan, Jared Trok, Andrew J. Wilson, Carlos F. Gould, Sam Heft-Neal, Marshall Burke, and Noah S. Diffenbaugh

Christopher Callahan.
E-mail: ccallah@iu.edu

This PDF file includes:

Figs. S1 to S8

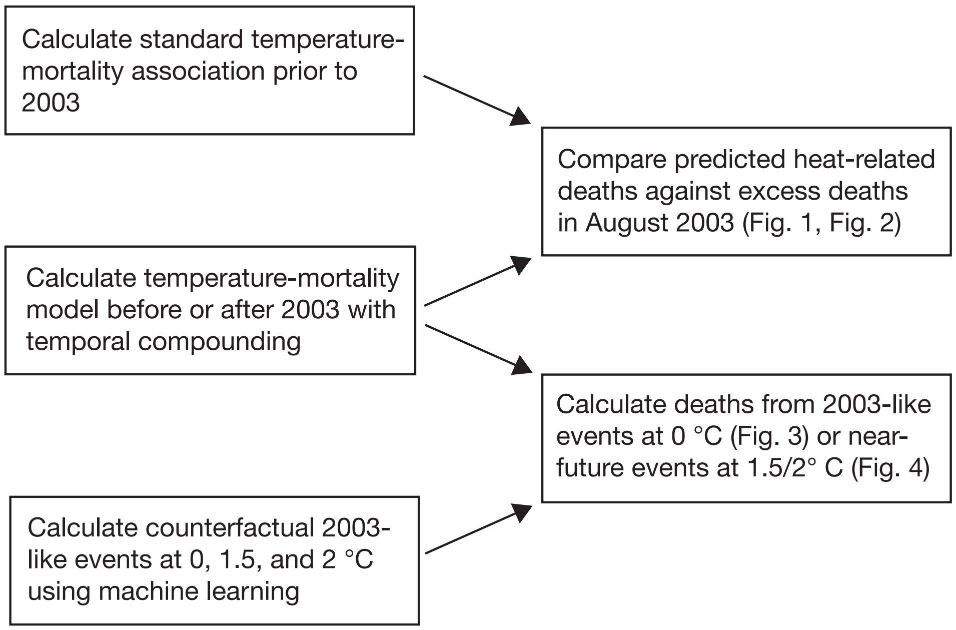

Fig. S1. Schematic of analysis. There are two broad components to our analysis. First, to empirically estimate exposure-response functions relating temperature and mortality and compare them to excess deaths during the August 2003 heat wave (Fig. 1, 2). Second, to calculate counterfactual events at pre-industrial or near-future global temperature levels, combined with these exposure-response functions to quantify the resulting mortality (Fig. 3, 4).

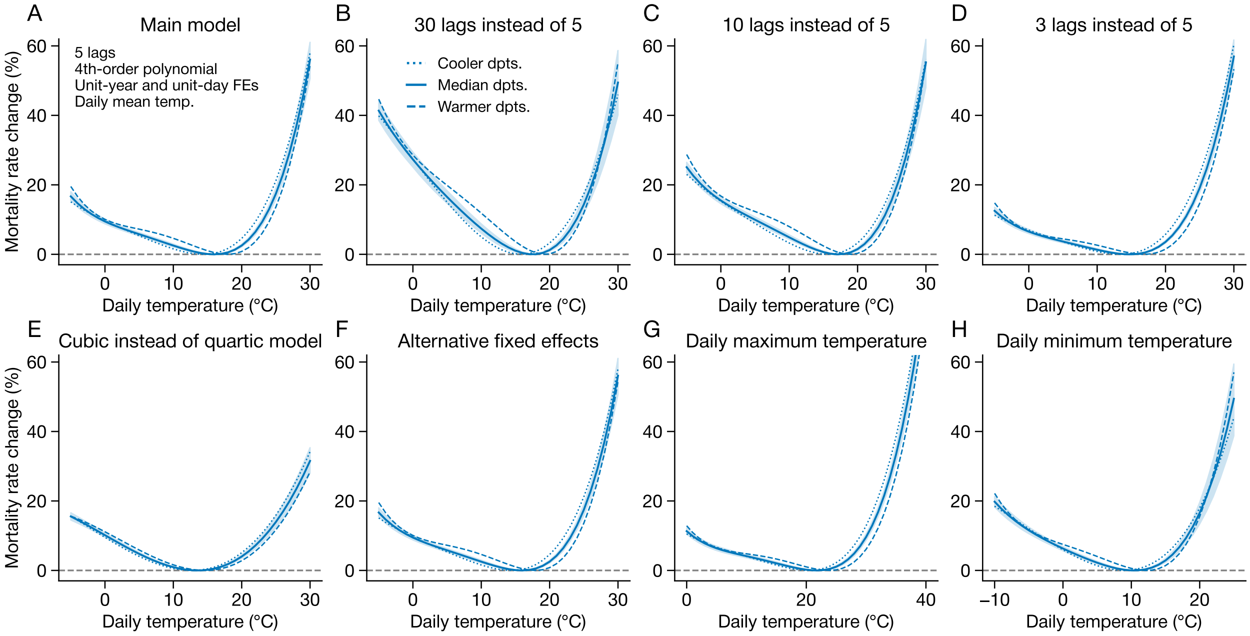

Fig. S2. Alternative exposure-response specifications. Each panel shows the cumulative response of mortality to temperature using a different regression specification. Panel (a) shows our main model using 5 lags, a 4th-order polynomial, unit-year and unit-day-of-year fixed effects, and daily mean temperature. Panel (b) increases the number of lags to 30, panel (c) increases the number of lags to 10, panel (d) reduces the number of lags to 3, panel (e) uses a cubic (3rd-order) instead of quartic model (4th-order), panel (f) uses separate unit, year, and day-of-year fixed effects, panel (g) uses daily maximum instead of mean temperature, and panel (h) uses daily minimum temperature. Each model includes an interaction with the department mean temperature; as in Fig. 1, we plot the responses at each of three terciles of department temperatures (see legend in panel b). Note the different x-axis ranges in (g) and (h) due to the different underlying ranges of the corresponding variables. 95% confidence intervals are shown as a shaded area on the response functions for median-temperature departments.

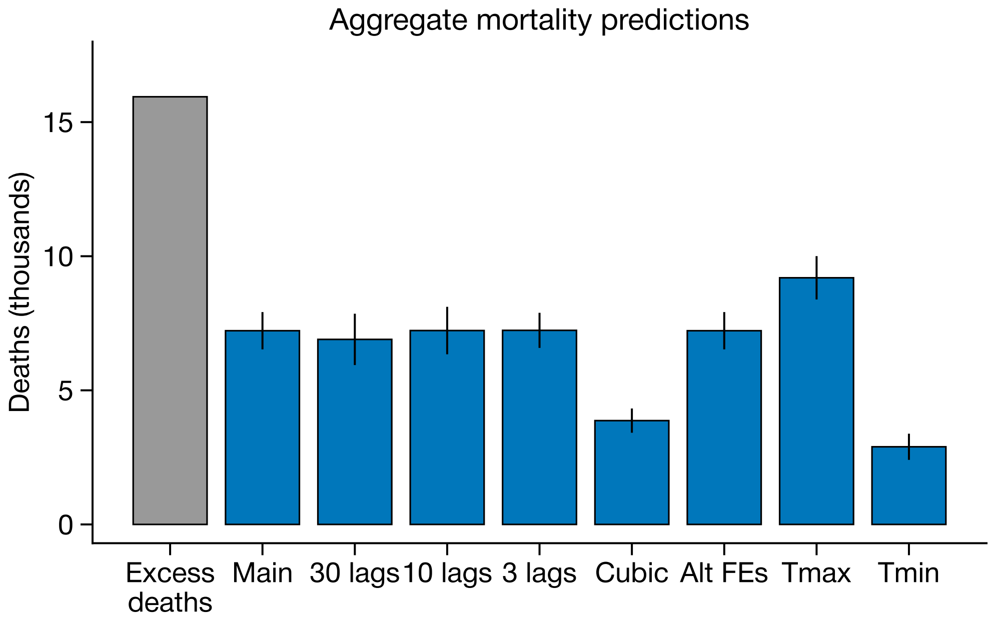

Fig. S3. Alternative mortality predictions for August 2003. As in Fig. 2d, but for the range of alternative specifications shown in Fig. S2.

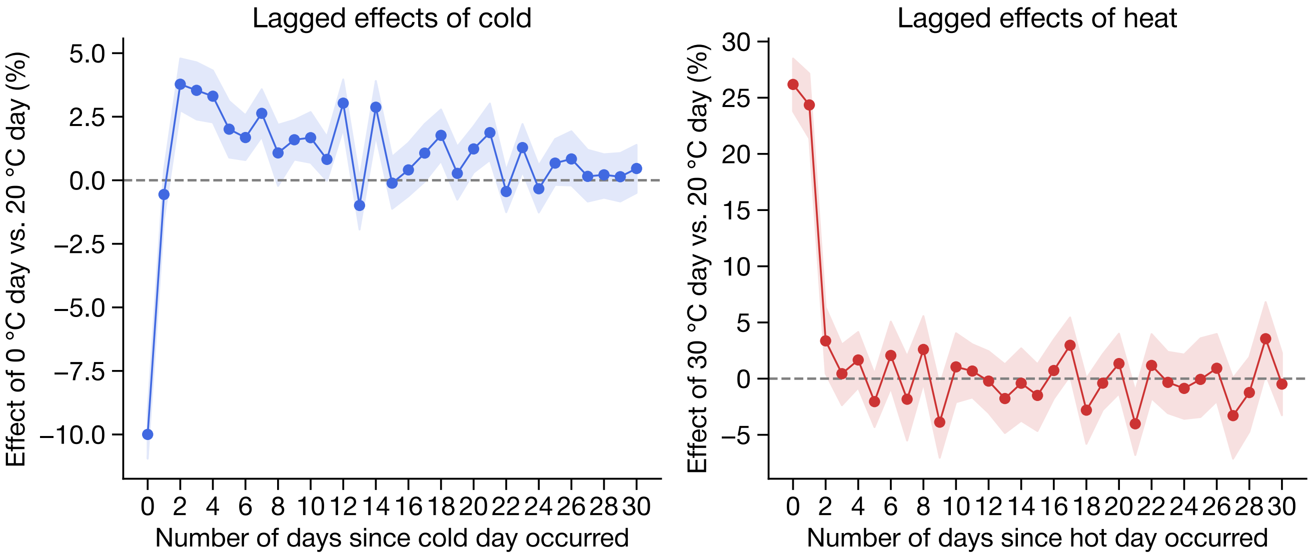

Fig. S4. Effect on mortality of cold and hot days over time. Cold days (left) are defined as 0 °C days compared to 20 °C days, and hot days (right) are defined as 30 °C days compared to 20 °C days. Dots show average across 500 bootstraps and shading shows 95% confidence interval. Lags are shown from a model that estimates contemporaneous (lag 0) effects and 30 lagged effects simultaneously. The model also includes an interaction with department-average climate as in the main analysis, and we present results for population-weighted average department temperature for simplicity.

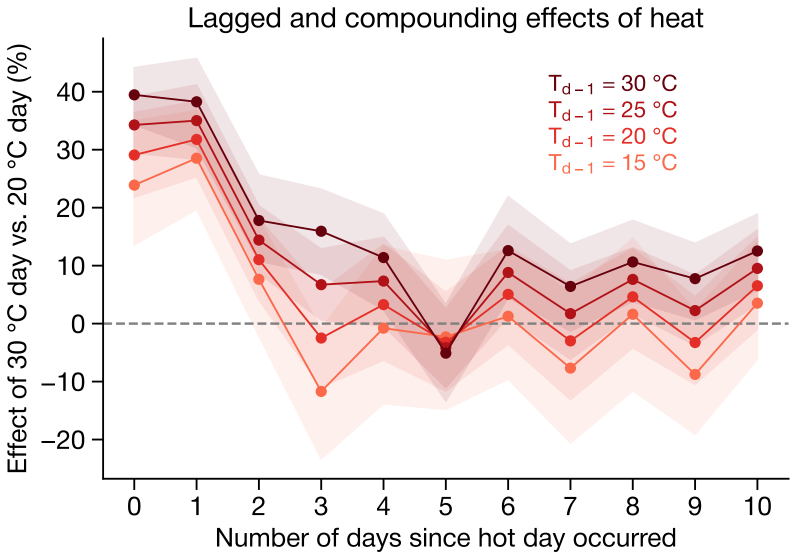

Fig. S5. Compounding heat effects across lags. Effect of a hot day on mortality (30 °C day vs. 20 °C day) across that day and the 10 days following it. Different lines show different responses conditioned on the temperature of the previous day from our main model of temporal compounding (Methods).

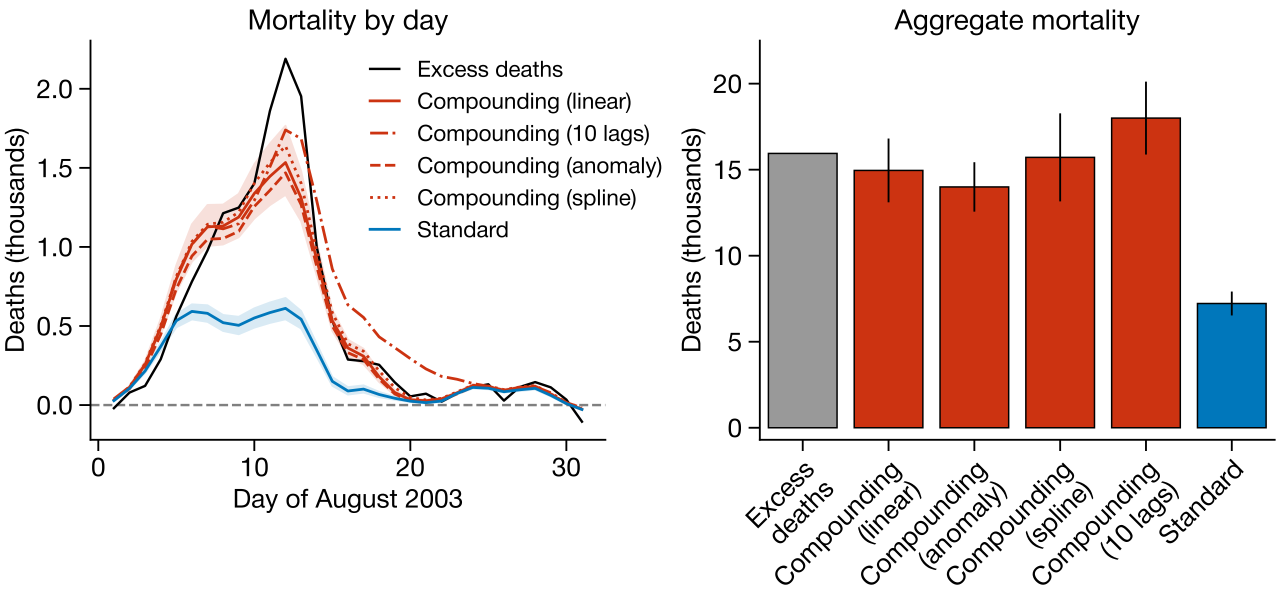

Fig. S6. Mortality predicted using alternative function forms for interaction. As in Fig. 2c and 2d, but adding results with a model that includes 10 lags, a model that interacts current-day temperatures with the previous day's temperature anomaly, and a model that uses a natural spline function in the previous day's temperature.

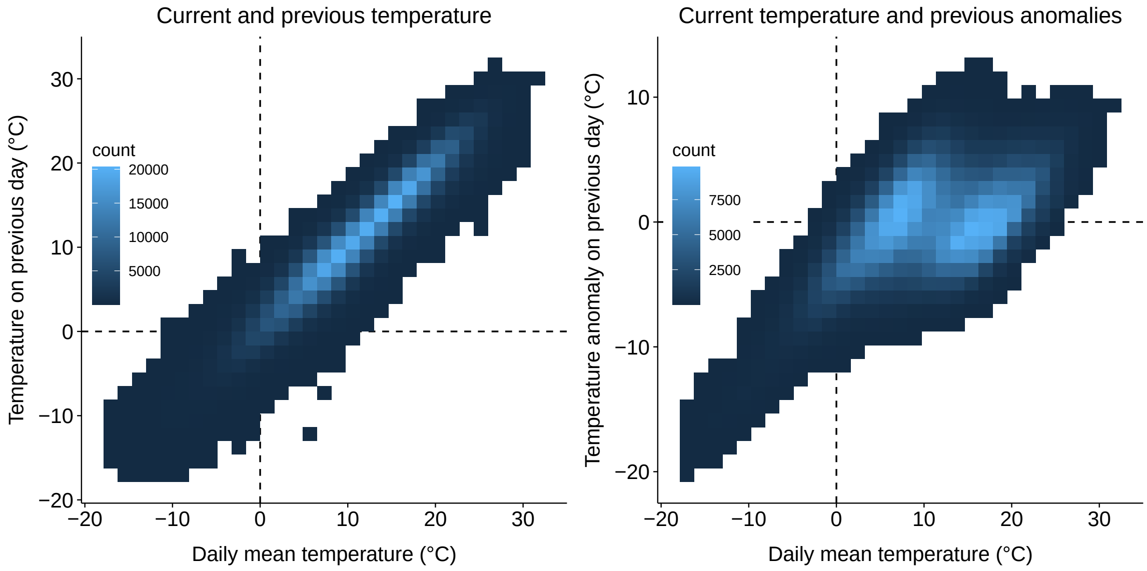

Fig. S7. Bivariate density of current and previous days' temperatures. Left: Relationship between temperature of current day (x-axis) and temperature of previous day (y-axis). Right: Relationship between temperature of current day (x-axis) and temperature *anomaly* of previous day (y-axis). Anomalies are calculated relative to day of year and department means. Coloring shows the number of observations in each two-dimensional bin.

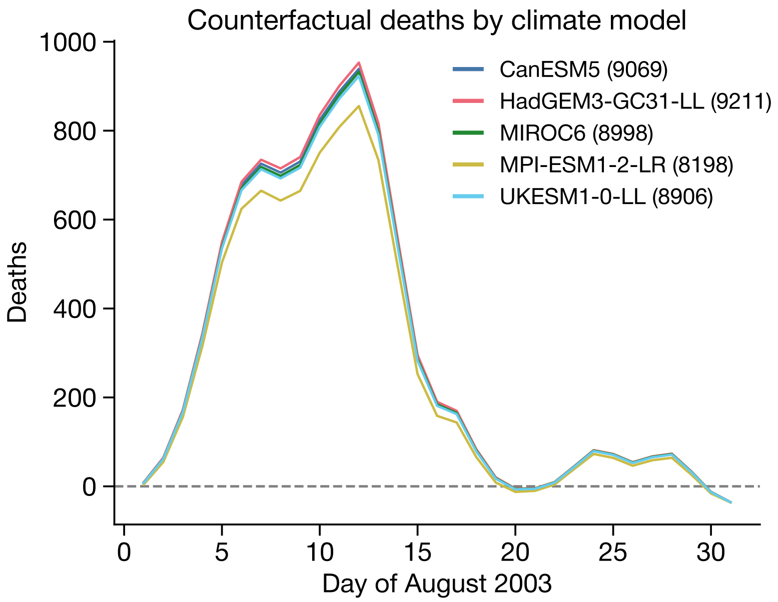

Fig. S8. Counterfactual mortality from two distinct climate models. As in Fig. 3b, but when separating the CNNs trained on the five different climate models (CanESM5, HadGEM3-GC31-LL, MIROC6, MPI-ESM1-2-LR, and UKESM1-0-LL). Lines show average counterfactual estimate when averaging across different random seeds used in training and different samples of the regression coefficients (Methods).
